# The clinical application of mesenchymal stromal cells in hematopoietic stem cell transplantation

**DOI:** 10.1186/s13045-016-0276-z

**Published:** 2016-05-18

**Authors:** Ke Zhao, Qifa Liu

**Affiliations:** Department of Hematology, Nanfang Hospital, Southern Medical University, 1838 Guangzhou Blvd North, Guangzhou, China

**Keywords:** Mesenchymal stromal cell, Hematopoietic stem cell transplantation, Engraftment failure, Graft-versus-host disease, Aplastic anemia, Infection, Relapse

## Abstract

Mesenchymal stromal cells (MSCs) are multipotent stem cells well known for repairing tissue, supporting hematopoiesis, and modulating immune and inflammation response. These outstanding properties make MSCs as an attractive candidate for cellular therapy in immune-based disorders, especially hematopoietic stem cell transplantation (HSCT). In this review, we outline the progress of MSCs in preventing and treating engraftment failure (EF), graft-versus-host disease (GVHD) following HSCT and critically discuss unsolved issues in clinical applications.

## Background

Mesenchymal stromal cells (MSCs), also called mesenchymal stem cells, are multipotent progenitors which were first described by Caplan and colleagues in 1991. They were first isolated from bone marrow (BM) and characterized by the ability to differentiate into adipogenic, chondrogenic, and osteogenic lineages [1–3]. Subsequently, a growing body of evidence suggests that MSCs can also be isolated from various tissue including umbilical cord blood (UCB), adipose tissue (AT), muscle, and dental pulp [4, 5]. Nowadays, due to the capacity to modulate immunological responses, support hematopoiesis, and repair tissue [6–8], MSCs have been widely used to treat immune-based disorders, such as Crohn’s disease, rheumatoid arthritis, diabetes, and multiple sclerosis [9–12]. Based on the animal experiments and clinical studies, the most successfully clinical application of MSCs is involved in hematological disease. In this review, we aim to elaborate the administration of MSCs in hematopoietic stem cell transplantation (HSCT) and aplastic anemia (AA), highlight the progress in MSCs functional features and the mechanisms of MSCs in clinical treatments.

### MSCs phenotype, characteristics, and expansion

MSCs are defined as non-hematopoietic, plastic-adherent, and self-renewing cells that are capable of differentiating into adipose, bone, and cartilage in vitro [13]. The International Society for Cellular Therapy (ISCT) has listed a panel of markers for identifying MSCs [13]. The minimum criteria of MSCs have been commonly used as follows:

1. Adherence to plastic in vitro culture
2. Surface antigens positive expression for CD105, CD73, CD90, and negative for markers including CD45, CD34, CD14 or CD11b, CD79α or CD19, and HLA-DR
3. Differentiation into osteoblasts, adipocytes, and chondrocytes in vitro

Subsequently, more cell-surface markers were discovered, such as stromal precursor antigen-1 (STRO-1), stage specific embryonic antigen-4 (SSEA-4), CD49a, CD271, CD146, and leptin receptor [14–19]. Apart from the emerging new markers, Edita Hamzic and colleagues showed that MSCs markers in AA patients differ from those in healthy people. Moreover, MSCs in AA patients exhibit significantly reduced hematopoiesis-supporting capacity [20]. Boome et al. also revealed that some specific biomarkers of MSCs in graft-versus-host disease (GVHD) patients express differently. The different expressions of these markers are good predictors for disease occurrence, resolution, and survival [21]. Therefore, it would be useful to explore more specific markers for diagnostic and prognostic applications.

In addition to the development of MSCs markers, exploration of efficient expansion of MSCs also plays an important role in clinical applications. In the fields of hematological diseases, MSCs are mainly derived from BM and UCB. However, MSCs only constitute less than 0.01 % overall cell population resident in BM, which is the major barrier for clinical usage [22]. An array of studies found that MSCs also rapidly lose their proliferation potential and multipotency through rounds of in vitro culture [23, 24]. Therefore, producing clinical-scale MSCs and maintaining their high proliferation potential and multipotency become very important. Recently, to increase the productivity of MSCs, researchers are applying different strategies by changing the culture media, optimizing culture density, eliminating hematopoietic stem cells and genetic modification [25–27].

### The function of MSCs

In general, MSCs possess the capacity to differentiate into various types of cells, home to sites of inflammation, repair tissue, modulate immune or inflammation response, and support hematopoiesis. Firstly, MSCs have been identified for their ability to differentiate into the bone, adipocytes, and cartilage [13]. With an increasing understanding of MSCs, investigators found that MSCs are capable of differentiating into all three germ layers [28–33]. And in some specific microenvironment, for example, injury, MSCs could differentiate into the lung epithelial cells in lung injury [34, 35] and into cardiomyocytes in myocardial infarction (MI) [36, 37]. Secondly, MSCs also have the ability to migrate to the sites of inflammation [38–40]. Using biofluorescence imaging technology, Joo and his colleagues monitored the delivery and biodistribution of red fluorescent protein (RFP)-labeled MSCs in GVHD model. They found that MSCs could first and rapidly home to the lungs, then migrate to other GVHD-injured organs, including the liver, esophagus, stomach, small intestine, and large intestine [41]. Furthermore, Hu et al. showed that CM-Dil-labeled MSCs can migrate to the thymus in aGVHD murine model [42]. Some researchers proposed that the degree of inflammation and different disease status might affect the distribution of MSCs. However, restricted by detecting method, tracing MSCs in deep target organs is hindered. Recent developed magnetic particle imaging allows researchers to accurately and quantitatively trace MSCs distribution [43]. Guided by the chemokines released from tissue or endothelial cells, MSCs migrate to specific sites and secrete large quantities of bioactive molecules to mediate repair [44, 45]. Lastly, MSCs also co-localize with hematopoietic stem cells (HSCs) in BM niche and produce factors recruiting HSCs and supporting hematopoiesis [46].

**Fig. 1:**
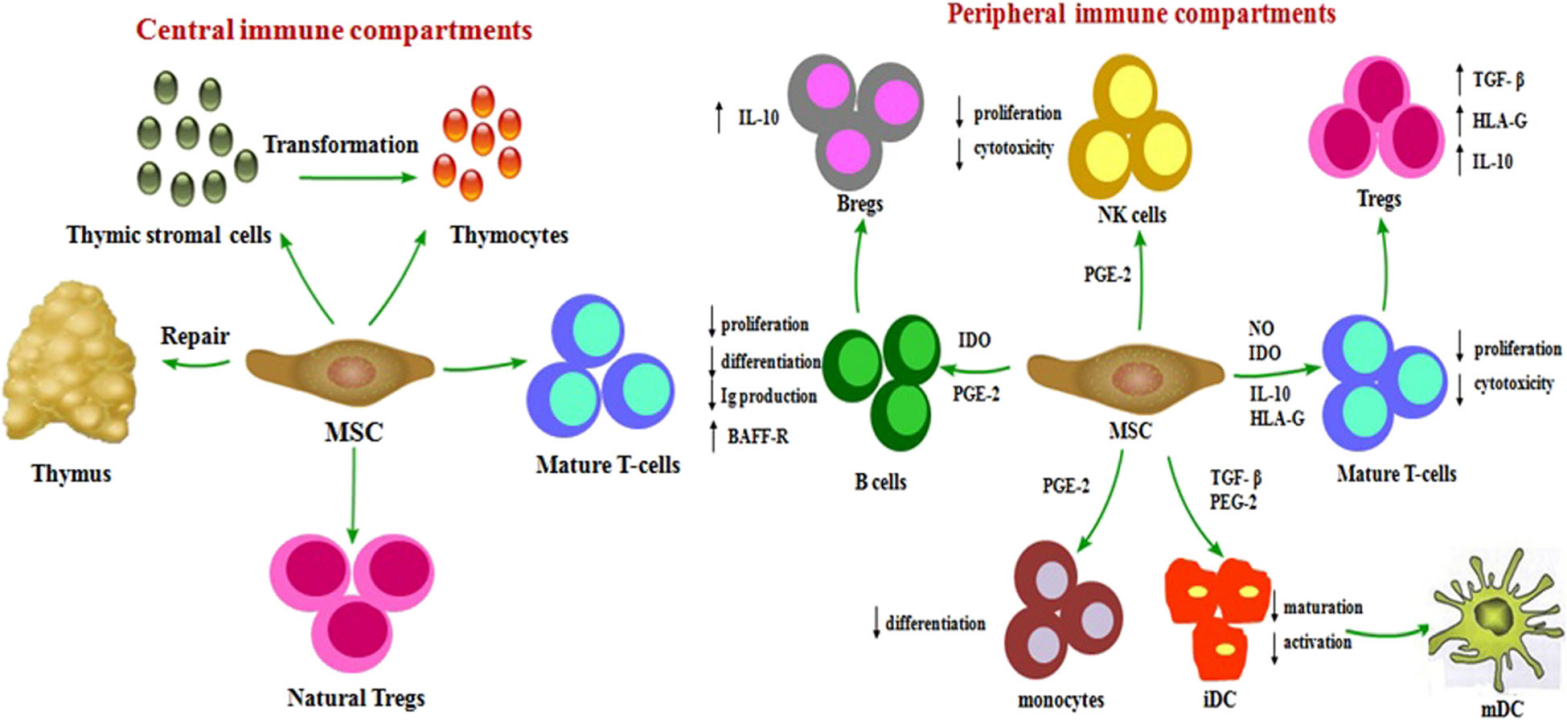
Immunomodulatory effects of MSC. MSCs exert immunomodulatory effects mainly through central and peripheral immune compartments. MSCs modulate central immune compartments by repairing damaged thymus, promoting T-cells maturation, inducing the proliferation of natural Tregs, and differentiating to thymocytes. MSCs modulate peripheral immune compartments, including interacting with various immune cells and secreting various soluble mediators involved in different microenvironment. They suppress T cell and B cell proliferation, induce the generation and proliferation of Tregs and Bregs, inhibit differentiation of precursors into DCs, suppress DCs maturation, and influence the function of NK cells

The highlight of MSCs function is its immune modulatory effects (Fig. 1). The mechanisms that MSCs regulate immune responses include interacting with various immune cells and secreting soluble mediators in different microenvironment [44, 45, 47, 48]. Initially, MSCs can express several adhesion molecules, including vascular cell adhesion molecule (VCAM)-1, intercellular cell adhesion molecule (ICAM)-1, and lymphocyte function-associated antigen (LFA)-3 involved in T-cell interactions, which result in the discovery of immunomodulatory properties of MSCs [49]. Further studies demonstrated that MSCs can not only suppress T-cell proliferation and activation, but also can regulate the differentiation of helper T (Th) cells [49, 50]. As an important subpopulation of T helper cells, regulatory T cells (Tregs) play a crucial part in inducing peripheral immune tolerance. MSCs can promote the generation of Tregs to prevent GVHD [51]. Moreover, MSCs are capable of inhibiting B-cell activation, proliferation, and the secretion of immunoglobulin (Ig) [52]. Our group recently documented that interleukin (IL)-10 produced by MSCs significantly increased CD5^+^ regulatory B cells (Bregs) production [53]. MSCs can increase the number of memory B lymphocytes and enhance B-cell activating factor receptor (BAFF-R) expression level on B lymphocytes [54]. MSCs also modulate immune responses by inhibiting differentiation of precursors into dendritic cells (DCs) as well as suppressing DCs maturation [55]. MSCs may inhibit DCs differentiation by producing IL-6 and macrophage colony-stimulating factor (M-CSF). And immature DCs generation in the presence of MSCs was significantly inhibited because MSCs induced the activation of T cells [56]. Finally, besides direct cell-to-cell contact mechanisms, MSCs also indirectly modulate immune response by producing many growth factors and cytokines, including transforming growth factor (TGF)-β, IL-6, prostaglandin E2 (PGE2), hepatocyte growth factor (HGF), indoleamine 2,3-dioxygenase (IDO), etc.

In the past, studies on the immunomodulatory effect of MSCs primarily focused on peripheral immune compartments. Recently, our group found that MSCs reduced the incidence and severity of cGVHD in aGVHD patients by improving thymic function [57]. Based on the role of MSCs in thymus development [4, 58] and our previous study, we proposed that MSCs could exert immunomodulatory effects through central immune compartments.

Besides, the most valuable discovery in recent years is the plasticity of MSCs in immune and inflammation regulation [48]. Traditionally, MSCs were considered as immunosuppression and anti-inflammation mediators by secreting high level of IDO, NO, PGE2, and TGF-β [48, 59]. However, researches recently found that MSCs are sensors of inflammation and are able to act as a pro-inflammatory or anti-inflammatory, immune enhancement, or immunosuppression phenotype depending on the involved inflammatory milieu [48]. The understanding of MSCs plasticity provides a new paradigm for MSCs-based cellular therapy and challenges MSCs clinical applications.

### Clinical applications of MSCs in HSCT

Currently, MSCs are widely used in hematological diseases, especially in HSCT, which mainly include promoting HSCs engraftment, treating engraftment failure (EF), poor graft function (PGF) and AA, and preventing and ameliorating GVHD.

### MSCs and hematopoiesis

Some studies indicated that MSCs play a vital role in modulating BM microenvironment and supporting hematopoiesis [46, 60, 61]. The capacity of MSCs to enhance engraftment has been proved in animal models [62, 63]. In clinical practice, MSCs are co-infused with HSCs to promote hematological engraftment and prevent EF and PGF. The first clinical trial on the use of MSCs promoting hematopoietic recovery is that autologous MSCs with HSCs were co-administered in breast cancer patients after high-dose chemotherapy [64]. Later, Lazarus et al. demonstrated that, when co-infused with HSCs, HLA-identical sibling donor derived MSCs were able to promote hematopoietic recovery in hematologic malignancy patients after HSCT [65]. In another pediatric study, MSCs were co-transplanted in 13 pediatric patients given UCB-derived HSCs. However, there was no significant difference between MSC-treated patients and histological controls in hematological engraftment [66]. Although there are still controversial on the efficacy of MSCs for hematopoietic engraftment, most published data demonstrated co-transplanting MSCs and HSCs were feasible and safe [60, 65, 67].

With regard to those patients developing to EF or PGF, MSCs could also be considered as a novel approach for these complications after HSCT. Taking EF after HSCT as an example, Meuleman et al. adopted MSCs without co-transplantation of HSCs to treat graft failure after HSCT [46]. Two of six EF patients achieved hematopoietic recovery after MSCs infusion, whereas four patients showed no response upon treatment. Based on Meuleman’s pilot study, we enrolled 22 patients, randomly assigned them into two groups and performed two cycles of treatments with MSCs or MSCs plus cord blood (CB), respectively [68]. After the first treatment cycle, 7 of 11 patients in MSC group had response, while 9 of 11 in MSC plus CB group (*P* = 0.635). There was a significant improvement in neutrophil reconstruction in MSC plus CB group compared with MSC group (*P* = 0.030). After the second treatment cycles, the overall response (OR) rate increased to 86.36 %. Therefore, our data indicated that these two strategies are both effective for EF treatment. Another study conducted in our group also showed that 17 out of 20 patients with PGF after allogeneic HSCT (allo-HSCT) responded to MSCs treatment, accompanied with an increased ratio of CD4^+^ to CD8^+^ T cells after MSCs administration [69].

Given the encouraging results of MSCs in EF and PGF treatments, the mechanism of MSCs for supporting hematopoiesis has been explored by several studies. In HSCT recipients, the hematopoietic microenvironment is damaged by chemotherapy, irradiation, and malignant hematological diseases [70, 71]. As a major constituent of BM microenvironment, MSCs can reconstitute the damaged stroma and secrete an array of hematopoietic cytokines, including IL-6, IL-7, IL-8, IL-11, Flt-3 ligand, and stem cell factor (SCF) to promote self-renew and differentiation of HSCs [46, 61]. MSCs can also improve hematopoiesis by modulating the inflammatory microenvironment and T-cell subtypes, which reduce the probability of graft rejection [60]. In addition, some studies found that MSCs promote hematopoietic recovery in AA patients. Our study also observed that six of 18 refractory AA patients (33.3 %) achieved OR after MSCs treatment, which was significantly higher than those in historic control cohort (5.56 %) [72]. After MSCs administration, MSC-treated patients exhibited a higher Tregs proportion. Thus, MSCs might promote AA patients hematopoietic recovery by inducing the generation of Tregs. Nonetheless, Diego V et al. showed that infusion of MSCs have no effect in refractory or relapsed AA patients in their clinical trial [73]. Some studies found that MSCs in AA patients presented distinctive markers, with decreased proliferative and haemopoiesis capacity [20]. Thus, MSCs as a therapeutic strategy for patients with AA are still controversial. Whether the characteristics of inherent MSCs in EF and PGF patients will change need to be further explored.

### MSCs and GVHD

GVHD remains the common and life-threatening complication limiting the widespread use of allo-HSCT, as GVHD associates with a high mortality and morbidity [74–77]. To date, new therapeutic agents have been made in GVHD prophylaxis. The efficacy of MSCs for GVHD prophylaxis varies in different studies [65, 78, 79]. Lazarus HM et al. reported that 28 % of patients developed aGVHD after co-infusion of MSCs with HSCs, while the incidence of aGVHD in patients who received only HSCs was 56 % in the historic control group [65]. Another study in Belgium investigated that MSCs given 30–120 min before peripheral blood stem cells (PBSCs) infusion significantly decreased the incidence of aGVHD [78]. Recently, patients in a prospective clinical trial were randomly divided into standard GVHD prophylaxis group and standard GVHD prophylaxis combining with MSCs group. MSCs were administered when the blood counts indicated recovery. The data supported that MSCs administration for aGVHD prophylaxis was effective and safe [80]. Although co-transplanted MSCs with HSCs to some extent decreased the incidence of aGVHD, most studies indicated that no statistical significance was shown in comparison with the historical control group.

Distinguished from the prevention of GVHD, MSCs application in the fields of aGVHD treatments achieved great success. Since Le Blanc et al. first reported that BM-derived MSCs rescued a pediatric patient experiencing grade IV refractory aGVHD [81], a number of studies have been performed to investigate the effect of MSCs for aGVHD treatment [57, 81–83]. Although the results are still controversial, most prospective and retrospective studies suggest that MSCs are effective to treat aGVHD. A large multicenter study of BM-derived MSCs for treating steroid-resistant aGVHD from the European Group for Blood and Bone Marrow Transplantation Mesenchymal Stem Cell Consortium showed that 30 of 55 patients had a complete response (CR) and nine showed partial response (PR), and the OR rate was 70.9 % [82]. Another large-scale, single-arm, prospective multicenter study enrolled pediatric steroid-refractory aGVHD patients from seven countries [83]. MSCs were given at a dose of 2 × 10^6^ cells/kg twice a week for four consecutive weeks. The response rate of GVHD target organs was respectively 58.5 % for the gastrointestine, 75.6 % for the skin, and 44.4 % for the liver. OR rate of refractory aGVHD patients treated with MSCs was 61.3 %. In our study, we designed a prospective study dividing refractory aGVHD patients to MSC group and non-MSC group. Combined with pre-existing aGVHD treatment, MSCs were intravenously infused once a week until aGVHD got CR or MSCs had been administered for a total of eight doses. Our results showed that OR rate was 75 % in MSC group, comparing with 42.1 % in non-MSC group (*P* = 0.023). Interestingly, we also found that the incidence of cGVHD decreased in MSC group compared with non-MSC group [57]. However, an American clinical trial evaluated the effect of an industrial MSCs product (Prochymal) and found that industrial MSCs failed to achieve a significant increase of CR rate in steroid-resistant GVHD patients compared with placebo [84]. Another study in Germany also showed a negative response to MSCs treatment in steroid-resistant aGVHD patients. MSCs were isolated from third-party donors and expanded in platelet lysate-containing medium. The OR rate and overall survival (OS) were not significantly different from those historical cohorts without MSCs treatment [85]. The patients in German study were old people who experienced more severe grade of aGVHD and extensive organ involvement. Therefore, it has been taken into consideration that the effects of MSCs for treating refractory aGVHD vary in different clinical trials, target organs, and even individuals.

MSCs for aGVHD treatments have been paid extensive attention, but the efficacy of MSCs for cGVHD has rarely been reported. As an autoimmune disorder, cGVHD has distinct manifestations from those of aGVHD [86]. Initially, a few studies showed that only limited and transient benefit could be observed in cGVHD patients after MSCs administration [87, 88]. Nonetheless, significant improvement has been reported by Weng et al. in refractory cGVHD patients after MSCs administration [89]. Fourteen of 19 refractory cGVHD patients receiving BM-derived MSCs treatment got OR, and the OR rate was 73.6 %. Consistent with Weng’s report, our group showed that 20 of 23 refractory cGVHD patients had a CR or PR in a 12-month follow-up study [53]. The most obvious improvements in cGVHD target organs were the skin, oral mucosa, and liver. Forcing on some specific cGVHD organs, Zhou et al. showed that MSCs were administered in four patients with sclerodermatous cGVHD (ScGVHD) by intra-BM injection [90]. Correspondingly, symptoms gradually improved in all four patients. Moreover, our group recently observed that the treatment of MSCs for bronchiolitis obliterans syndrome (BOS) was effective, which is the only single patho-pneumonic manifestation of cGVHD according to the National Institutes of Health (NIH) [91, 92]. Fifty-three patients with refractory BOS were enrolled in our prospective study, including 29 patients in MSC group and 24 patients in non-MSC group. The OR rate in MSC group (75.9 %) was significantly higher than that in non-MSC group (16.7 %). And the efficacy of MSCs to refractory BOS was significantly related to the severity of pulmonary function [92].

Although current studies demonstrate the encouraging treatment effects of MSCs for cGVHD patients, the responsiveness of MSCs to aGVHD patients is superior to that of cGVHD [80]. The differences between the treatments for aGVHD and cGVHD might relate to the different mechanisms of MSCs for a/cGVHD treatments. It is well known that T cells play crucial roles in the development of aGVHD. Joo et al. reported that MSCs could migrate to the damaged organs in aGVHD murine model, such as the lungs, gut, liver, skin, thymus, and lymph nodes [41]. As a sensor in local microenvironment, MSCs can sensitively receive inflammation signals to exhibit their immune and inflammation regulation effects by indirectly secreting various cytokines or chemokines and directly interacting with peripheral immune cells, inducing the generation and proliferation of Tregs [44, 45, 47, 48]. Moreover, our group found that MSCs can ameliorate aGVHD through central immune compartments. MSCs improve thymic output function and reconstruct damaged thymic structure which induced a long-term immune tolerance [57, 58]. And MSCs modulate cGVHD through influencing the function of B lymphocytes. By analyzing the clinical samples of cGVHD patients, our group demonstrated that MSCs promote B-lymphocyte reconstruction and sustain B-lymphocyte homeostasis by increasing naive and memory B-cell subsets in responsive cGVHD patients, and modulating plasma BAFF levels and BAFF-R expression on B lymphocyte [54]. In addition, our further study also found that MSCs promote regulatory CD5^+^ B cells (Bregs) proliferation in responsive cGVHD patients [53].

Nowadays, an increasing number of researchers pay their attention to the therapeutic differences of MSCs treatment in GVHD individuals. So, it will be meaningful to know what kinds of patients may benefit from MSCs treatment. GVHD biomarker profiling plays an indicative role on occurrence, resolution, or survival in the context of GVHD come from the analysis of MSCs treatment. Boome et al. first performed a prospective study to explore the relationship between clinical outcomes and the level of soluble biomarkers. Forty-eight steroid-refractory aGVHD patients with MSCs treatment were enrolled in this study. The results showed that the 1-year OS in MSC-treated patients can be predicted by soluble biomarkers, including IL-2 receptor α, tumor necrosis factor (TNF) receptor 1, HGF, regenerating islet-derived protein 3α, and elafin [21]. In another study, tumorigenicity 2 (ST2) has been reported as a strong predictive marker for non-response to GVHD therapy [93]. Therefore, further prospective clinical trials are needed to discover more biomarkers to guide MSCs treatment.

### MSCs and infection, relapse

Currently, it is still controversial on whether MSCs increase the incidence of infection and tumor recurrence. In the past, some studies showed that MSCs increased the risk of infections and tumor relapse by suppressing T-cell response and secreting some cytokines, including VEGF and IL-6 [94–96]. Ning et al. reported that MSCs increased the risk of tumor relapse in patients co-transplanted HSCs with MSCs to prevent GVHD [95]. However, our studies showed that the incidence of infection and tumor relapse did not increase after MSCs treatment for aGVHD, EF, and PGF [57, 69], which was consistent with other recent studies [40, 57, 68, 69, 97–99].

Another interesting discovery is that MSCs possess the potential to control bacteria, viruses, and protozoal parasites infections. Jeffery J et al. proposed that MSCs exerted their antimicrobial ability by direct effects on the pathogen or indirect effects through secreting soluble factors and enhancing anti-inflammation function of immune cells [100]. Our group also showed that human MSCs pre-stimulated with IFN-γ could inhibit the growth of *Toxoplasma gondii* via upregulation of GBP-1 [101]. For antivirus effect, MSCs decrease the replication of cytomegalovirus (CMV) by secreting cytokine IDO [102]. MSCs cannot suppress the production of viral-specific T cells, but can inhibit the function of alloantigen and mitogen-induced T cells [99]. To test the efficacy of MSCs as a novel antimicrobial therapy, the first clinical trial was applied in sepsis-induced acute lung injury model [103]. The efficacy of MSCs for infections following HSCT needs to be further explored.

### MSCs source, dosage, and therapeutic schedule

There are an increasing number of clinical trials on MSCs administration in HSCT. However, the efficacy of MSCs treatment varied in different clinical trials, diseases, target organs, and even different individuals. An array of factors might influence the effects of MSCs treatment, such as source of MSCs, dosage to be infused, therapeutic schedule, and the route and timing of MSCs administration. Firstly, it is important to recognize that MSCs are poorly defined by phenotypical or functional features. No standard has been established for clinical grade MSCs manufacture. Nowadays, MSCs products are derived from different tissue (BM, AT, UCB, or placental), different donors (autologous, donor derived, or third-party), different laboratories (industrial or manufactured by academic centers), and are cultured and expanded from different media and conditions [4, 5, 84, 104]. Subsequently, the number of MSCs infusion has ranged from 0.4 × 10^6^ to 10 × 10^6^/kilogram of body weight [57, 82, 105]. Usually, the widely accepted dosage of MSCs administration is approximately 1 × 10^6^/kilogram of body weight. The therapeutic schedule has also been designed as single or repeated doses of MSCs in different intervals. Recently, a study showed that the characteristics of individuals, for example, the immune and inflammation microenvironment in vivo, might influence the effects of aGVHD patients [21]. Thus, the timing of MSCs infusion is also very important. In addition, the route of MSCs administration should be considered. To date, intravenous injection is still the main route for the delivery of MSCs for hematologic disorders in human trials and animal models. Another possibility is to administrate MSCs by intra-arterial infusion, which was reported by Arima et al. in limited three steroid refractory aGVHD patients. MSCs were infused into mesenteric arteries for gut GVHD and hepatic artery for hepatic GVHD via selective angiography. But the study was stopped due to unsatisfied GVHD response [106]. Zhou et al. gave MSCs directly into the BM by the anterosuperior iliac spine to treat ScGVHD, and all the patients had significant improvements in their GVHD symptoms [90]. However, whether intra-BM infusion improves the efficacy of MSCs treatment requires further study. The optimization of therapeutic procedure for MSCs clinical application also needs large scale and prospective studies.

## Conclusions

Nowadays, the most successfully clinical application of MSCs is involved in HSCT and AA. The efficacy of MSCs treatment varies in different studies, but the majority of studies show that MSCs are promising cellular therapy. However, there are still some hurdles to overcome. Firstly, standardized process of MSCs production has not been established, including the source of MSCs, culture media, and passage, etc. How to establish an efficient expansion system to satisfy MSCs clinical need, meanwhile maintain the high proliferation potential and multipotency needs further study. Secondly, MSCs therapeutic strategies varied in different clinical studies for treatments of hematological disorders. And advances in personalized medicine showed that the efficacy of MSCs treatment might be related with the individual immune and inflammation microenvironment. The optimized route, dose, frequency, and treatment interval of MSCs administration require better understanding of the mechanisms of MSCs treatment. Moreover, MSCs might promote tumor growth and progression, because they have the ability to suppress immune response and secrete some mediators driving angiogenesis in theory [95]. However, MSCs actually exert bidirectional effects on tumor regulation. They can inhibit tumors by activating tumor-suppression signaling pathways in a recent study [40]. Taken together, future success of MSCs therapy will depend on rational optimization of therapeutic strategies in conjunction with an adequate understanding of therapeutic mechanisms.

## Abbreviations

AA: aplastic anemia
aGVHD: acute GVHD
allo-HSCT: allogeneic HSCT
AT: adipose tissue
BAFF-R: B-cell activating factor receptor
BM: bone marrow
BOS: bronchiolitis obliterans syndrome
Bregs: regulatory B cells
CB: cord blood
cGVHD: chronic GVHD
CMV: cytomegalovirus
CR: complete response
DCs: dendritic cells
EF: engraftment failure
GVHD: graft-versus-host disease
HGF: hepatocyte growth factor
HSCs: hematopoietic stem cells
HSCT: hematopoietic stem cell transplantation
ICAM: intercellular cell adhesion molecule
IDO: indoleamine 2,3-dioxygenase
Ig: immunoglobulin
IL: interleukin
ISCT: International Society for Cellular Therapy
LFA: lymphocyte function-associated antigen
M-CSF: macrophage colony-stimulating factor
MI: myocardial infarction
MSCs: mesenchymal stromal cells
NIH: the National Institutes of Health
OR: overall response
OS: overall survival
PBSCs: peripheral blood stem cells
PGE2: prostaglandin E2
PGF: poor graft function
PR: partial response
RFP: red fluorescent protein
SCF: stem cell factor
ScGVHD: sclerodermatous cGVHD
SSEA-4: stage-specific embryonic antigen-4
ST2: suppression of tumorigenicity 2
STRO-1: stromal precursor antigen-1
TGF: transforming growth factor
Th: helper T
TNF: tumor necrosis factor
Tregs: regulatory T cells
UCB: umbilical cord blood
VCAM: vascular cell adhesion molecule
